# Killing Two Birds with One Stone: Discovery of Dual Inhibitors of Oxygen and Fumarate Respiration in Zoonotic Parasite, Echinococcus multilocularis

**DOI:** 10.1128/aac.01428-22

**Published:** 2023-02-22

**Authors:** Shigehiro Enkai, Hirokazu Kouguchi, Daniel Ken Inaoka, Tomoo Shiba, Masahito Hidaka, Hiroyuki Matsuyama, Takaya Sakura, Kinpei Yagi, Kiyoshi Kita

**Affiliations:** a Department of Pediatrics, Teikyo University School of Medicine, Tokyo, Japan; b School of Tropical Medicine and Global Health, Nagasaki University, Nagasaki, Japan; c Department of Infectious Diseases, Hokkaido Institute of Public Health, Sapporo, Hokkaido, Japan; d Department of Molecular Infection Dynamics, Shionogi Global Infectious Diseases Division, Institute of Tropical Medicine (NEKKEN), Nagasaki University, Nagasaki, Japan; e Department of Biomedical Chemistry, Graduate School of Medicine, The University of Tokyo, Tokyo, Japan; f Department of Applied Biology, Graduate School of Science Technology, Kyoto Institute of Technology, Kyoto, Japan; g Laboratory of Parasitology, Department of Disease Control Faculty of Veterinary Medicine, Hokkaido University, Sapporo, Hokkaido, Japan; h Department of Host-Defense Biochemistry, Institute of Tropical Medicine (NEKKEN), Nagasaki University, Nagasaki, Japan

**Keywords:** *Echinococcus multilocularis*, ascofuranone, structure-activity relationship, mitochondrial complex II, fumarate respiration, drug development, dual inhibitors

## Abstract

Ascofuranone (AF), a meroterpenoid isolated from various filamentous fungi, including *Acremonium egyptiacum*, has been reported as a potential lead candidate for drug development against parasites and cancer. In this study, we demonstrated that AF and its derivatives are potent anthelminthic agents, particularly against Echinococcus multilocularis, which is the causative agent of alveolar echinococcosis. We measured the inhibitory activities of AF and its derivatives on the mitochondrial aerobic and anaerobic respiratory systems of E. multilocularis larvae. Several derivatives inhibited complex II (succinate:quinone reductase [SQR]; IC_50_ = 0.037 to 0.135 μM) and also complex I to III (NADH:cytochrome *c* reductase; IC_50_ = 0.008 to 0.401 μM), but not complex I (NADH:quinone reductase), indicating that mitochondrial complexes II and III are the targets. In particular, complex II inhibition in the anaerobic pathway was notable because E. multilocularis employs NADH:fumarate reductase (fumarate respiration), in addition to NADH oxidase (oxygen respiration), resulting in complete shutdown of ATP synthesis by oxidative phosphorylation. A structure-activity relationship study of E. multilocularis complex II revealed that the functional groups of AF are essential for inhibition. Binding mode prediction of AF derivatives to complex II indicated potential hydrophobic and hydrogen bond interactions between AF derivatives and amino acid residues within the quinone binding site. *Ex vivo* culture assays revealed that AF derivatives progressively reduced the viability of protoscoleces under both aerobic and anaerobic conditions. These findings confirm that AF and its derivatives are the first dual inhibitors of fumarate and oxygen respiration in E. multilocularis and are potential lead compounds in the development of anti-echinococcal drugs.

## INTRODUCTION

Alveolar echinococcosis is a chronic, progressive, and life-threatening parasitic disease that is caused by the larval metacestode stage of Echinococcus multilocularis. This parasite is a public health concern throughout the Northern Hemisphere. Currently, albendazole is the only available chemotherapy for alveolar echinococcosis and shows insufficient efficacy (1, 2). Therefore, echinococcosis has been designated as a neglected tropical disease, and the development of novel drugs is urgently required (3). As such, we focused on the mitochondrial respiratory chain of E. multilocularis as a drug target, and reported that atovaquone, a potent inhibitor of oxygen respiration that targets mitochondrial complex III (quinol:cytochrome *c* reductase), shows a synergic effect *ex vivo* and also *in vivo* when administered in combination with albendazole (4). Despite increasing evidence that the quinone binding sites of the mitochondrial respiratory complexes of the parasite are a potential target space, development has been challenging due to the plasticity of mitochondrial respiration. This plasticity allows the parasite to (i) alternate between oxygen and fumarate respiration, and (ii) rapidly adapt to the presence of individual respiratory inhibitors. In other words, one respiration mechanism being active is sufficient to maintain the electrochemical gradient that is required to synthesize ATP by oxidative phosphorylation, and facilitate parasite survival.

**FIG 1:**
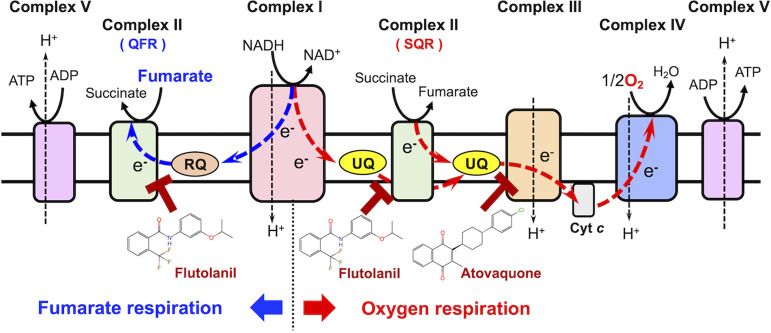
Overview of the mitochondrial respiratory chain of E. multilocularis. Fumarate respiration (NADH:fumarate reductase system) involves complex I, rhodoquinone (RQ), and complex II (quinol:fumarate reductase, QFR). In this system, electrons from NADH are transferred to RQ through complex I, and then transferred to fumarate by the QFR activity of complex II. An electrochemical gradient is maintained by the activity of complex I, and ATP is generated by oxidative phosphorylation (complex V) even under hypoxic conditions. Oxygen respiration is performed by complexes I, II, III, and IV, with ubiquinone (UQ) acting as an electron carrier between complexes I/II and III, and cytochrome *c* (Cyt *c*) between complexes III and IV. Complex II transfers electrons from succinate to UQ, acting as a succinate:quinone reductase (SQR). At the end of oxygen respiration, the electrons from NADH and succinate are used to reduce oxygen molecules to form water. Flutolanil and atovaquone are quinone binding site inhibitors of complexes II and III, respectively.

Oxygen respiration is driven by the activities of complexes I, II, III, and IV, which make up the mitochondrial electron transport chain (ETC). Complexes I (NADH:quinone oxidoreductase) and II (succinate:quinone reductase, SQR) transfer electrons from NADH and succinate, respectively, to complex III via the ubiquinone pool (Fig. 1). The reducing equivalents from the ubiquinone pool are transferred by complex III to complex IV via cytochrome *c*. At the end of the ETC, electrons are finally transferred to dioxygen, resulting in the production of water. In the aerobic pathway, complex III of E. multilocularis has been reported as a feasible and promising drug target for echinococcosis, because the antimalarial atovaquone strongly inhibits complex III in E. multilocularis and restricts the growth of larval cysts *in vivo* (5). Fumarate respiration involves mitochondrial complexes I and II, and plays an important role in the survival of E. multilocularis under hypoxic conditions in the host (6). Fumarate respiration is composed of complex I, low-potential electron mediator rhodoquinone (RQ), and the reverse reaction catalyzed by complex II (quinol:fumarate reductase [QFR]). The reducing equivalent from NADH is first transferred to RQ by complex I, and then from the reduced RQ to fumarate via the QFR activity of complex II, producing succinate (Fig. 1).

The electrochemical gradient that is formed during oxygen respiration is maintained by the proton pump activity of complexes I, III, and IV and is used by complex V to synthesize ATP. Although complex I becomes the sole respiratory complex to maintain the electrochemical gradient during fumarate respiration, it is capable of synthesizing ATP even in the absence of oxygen. Moreover, considering the internal hypoxic condition of the hydatic cyst, fumarate respiration is likely predominant in E. multilocularis protoscoleces, and both complex I and II have been suggested as drug targets (6, 7). In fact, quinazoline derivatives that inhibit E. multilocularis complex I effectively kill protoscoleces in *ex vivo* assays (6). Given that quinazoline and its derivatives also inhibit mammalian complex I, the identification of selective inhibitors of parasite respiratory chain enzymes will be key in developing drugs for alveolar echinococcosis.

Ascofuranone (AF) is a meroterpenoid compound produced by filamentous fungi, including *Acremonium egyptiacum* (8, 9). AF has been reported to be an inhibitor of the quinone binding site of mitochondrial enzymes in various organisms, and it strongly inhibits the ubiquinol oxidase activity of Trypanosoma brucei mitochondrial alternative oxidase (TAO), an enzyme that is essential for parasite survival (10–13). Recent studies have shown that AF strongly inhibits the mitochondrial respiratory chain of Schistosoma mansoni and reduces the worm burden (14). Furthermore, AF and its derivatives do not inhibit mammalian respiratory chain complexes, although these compounds potently inhibit human dihydroorotate dehydrogenase (HsDHODH). This enzyme is the rate-limiting step of the pyrimidine *de novo* biosynthesis pathway, and inhibition of HsDHODH drastically reduces the viability of cancer cells, especially under tumor-microenvironment mimicking conditions (hypoxia and nutrient-deprivation) (15, 16). It has been reported that AF also suppresses the signaling pathways of invasion and migration in cancer cells (17, 18). Recently, the entire biosynthetic pathway of AF has been clarified in *A. egyptiacum* (19, 20); therefore, further manipulation of this strain could enable mass production of AF and its biosynthesis intermediates to perform *in vivo* experiments and evaluate its clinical applications.

Here, we report novel pharmacological findings of AF and its derivatives, which could contribute to the development of anti-echinococcal drugs as well as elucidate mitochondrial function in E. multilocularis. We examined the inhibitory activities of AF and its derivatives on the mitochondrial respiratory chain of E. multilocularis and evaluated their structure-activity relationship with complex II and their binding mode. In addition, we demonstrated the efficacy of AF derivatives against E. multilocularis protoscoleces in *ex vivo* assays under aerobic and anaerobic conditions.

## RESULTS

### Effects of AF derivatives on the mitochondrial respiratory chain of E. multilocularis.

Table 1 (see AF, D1 to 5) shows the inhibitory potency of AF and its derivatives against complexes I, II, and III. The NADH:ubiquinone reductase activity of complex I was not inhibited by AF or any of its derivatives. For the SQR activity of complex II, several derivatives showed inhibitory activity at low concentrations, IC_50_ = 0.088 to 0.135 μM. The NADH:cytochrome *c* reductase activity by complexes I and III, was potently inhibited by AF derivatives, with IC_50_ values of 0.008 to 0.274 μM. As complex I is not inhibited by AF or its derivatives, the target of inhibition is complex III. Notably, several AF derivatives inhibited both complexes II and III, although the inhibitory activity for each complex differed according to the structure of the compounds.

**TABLE 1:**
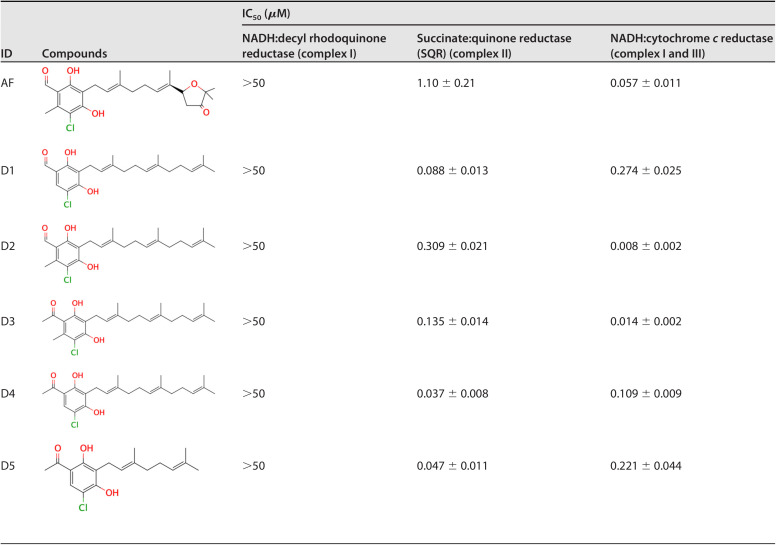
Inhibitory effect of ascofuranone and representative derivatives on E. multilocularis protoscoleces mitochondria

### Structure-activity relationship of AF and its derivatives against complex II (SQR) of E. multilocularis.

As the inhibition of complex II activity by AF and its derivatives can be examined directly, the structure-activity relationship of the compounds was examined. Structurally, AF is comprised of benzene, linker, and terminal groups (Fig. S1). Table 2 shows several AF derivatives with alterations in the benzene group that were evaluated (see AF, D5 to 11). Substitution of the aldehyde group (D7) increased the IC_50_ 2-fold compared to the acetyl group (D6), indicating that the 1-acetyl group is more favorable than the 1-aldehyde group for inhibiting SQR activity. When the acetyl group (D6) or 1-aldehyde group (D7) was changed to a methyl ester group (D9), the inhibitory activity was lost (IC_50_ > 10 μM), suggesting that substituents bulkier than an acetyl group are deleterious for the inhibition of SQR activity. Next, the contribution of the 5-Cl group was evaluated. A comparison between D7 and D8 revealed that the 5-Cl group is essential for the inhibitory activity. However, when the 6-methyl group was removed from the benzene ring, the inhibitory activity was increased by 2- to 3-fold (D5 and 6, AF and 10). In addition, when the 4-OH group in AF was changed to a methoxy group (as D11), the inhibitory activity was lost. The contribution of the 2-OH group for the inhibition of complex II could not be evaluated in this study because of difficulties in the synthesis of AF derivatives with substitutions only at this position.

**TABLE 2:**
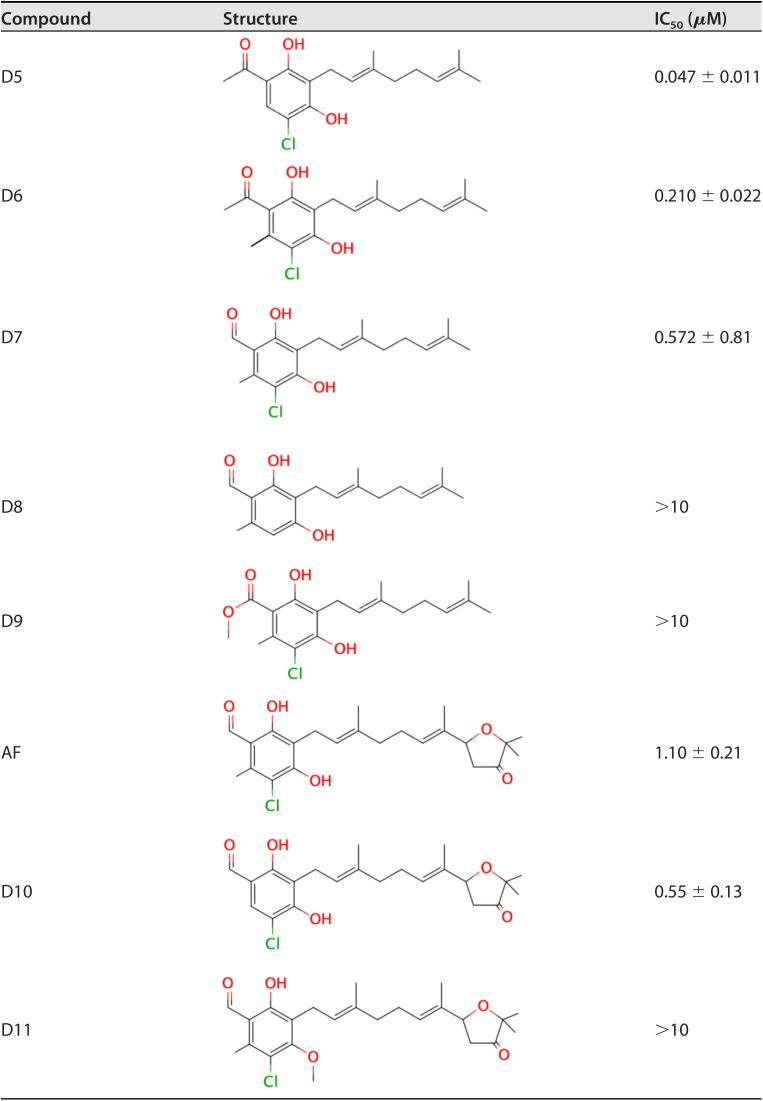
Inhibition of *E. multilocularis* SQR by derivatives with different substitutions on the benzene group

The effect of the linker length of AF derivatives on SQR inhibition was also evaluated. As shown in Table 3, the optimum linker lengths for potent inhibition were observed at C7, C9, and C10 (IC_50_ = 0.95, 1.2, and 1.2 μM, respectively). In contrast, compounds with either a short linker (C0, C3, and C5) or the longest one (C12) exhibited a remarkable decrease in potency. Modifications of the linker group were also evaluated (Table 4). Four types of linker chain were compared to clarify the function of the linker group. The IC_50_ values of compounds D12 (geranyl chain), D13 (two branched methyl groups), D14 (one branched methyl group), and D15 (linear alkyl chain) were 0.57, 0.28, 0.54, and 0.89 μM, respectively. The IC_50_ of D13 with two branched methyl groups was three times lower than that of D15, which had a linear alkyl chain.

**TABLE 3:**
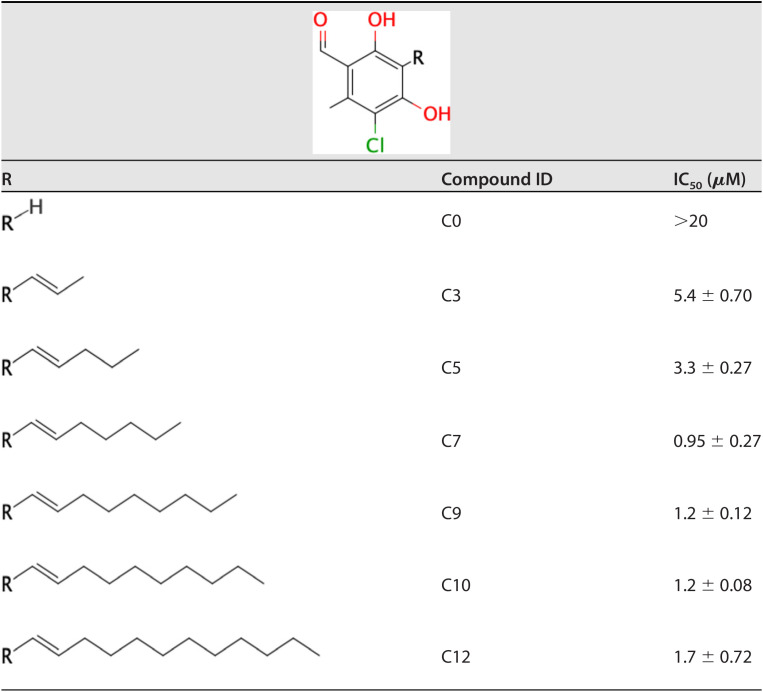
IC_50_ against E. multilocularis SQR of derivatives with various side chain lengths

**TABLE 4:**
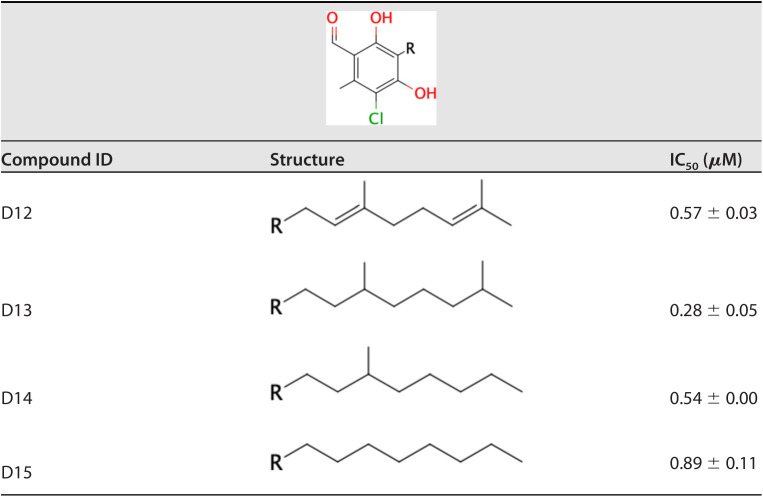
Inhibition of E. multilocularis SQR by different linker structures

AF derivatives possessing changes in the terminal group were also evaluated (Table 5). Changing the furanone ring to other groups, such as in D2, 16, and 17, resulted in IC_50_ values decreasing to 0.30, 0.25, and 0.48 μM, respectively, compared with an IC_50_ of 1.1 μM for AF. However, IC_50_ was increased in several groups, such as D18 and 19.

**TABLE 5:**
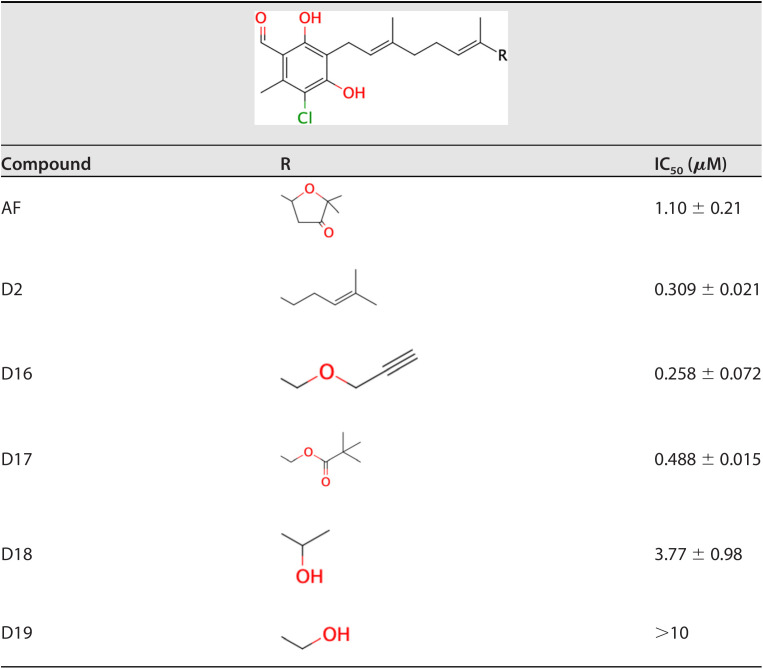
E. multilocularis SQR inhibition by furanone ring-substituted derivatives

### Structural insights into the inhibition mechanism of complex II by AF derivatives.

**FIG 2:**
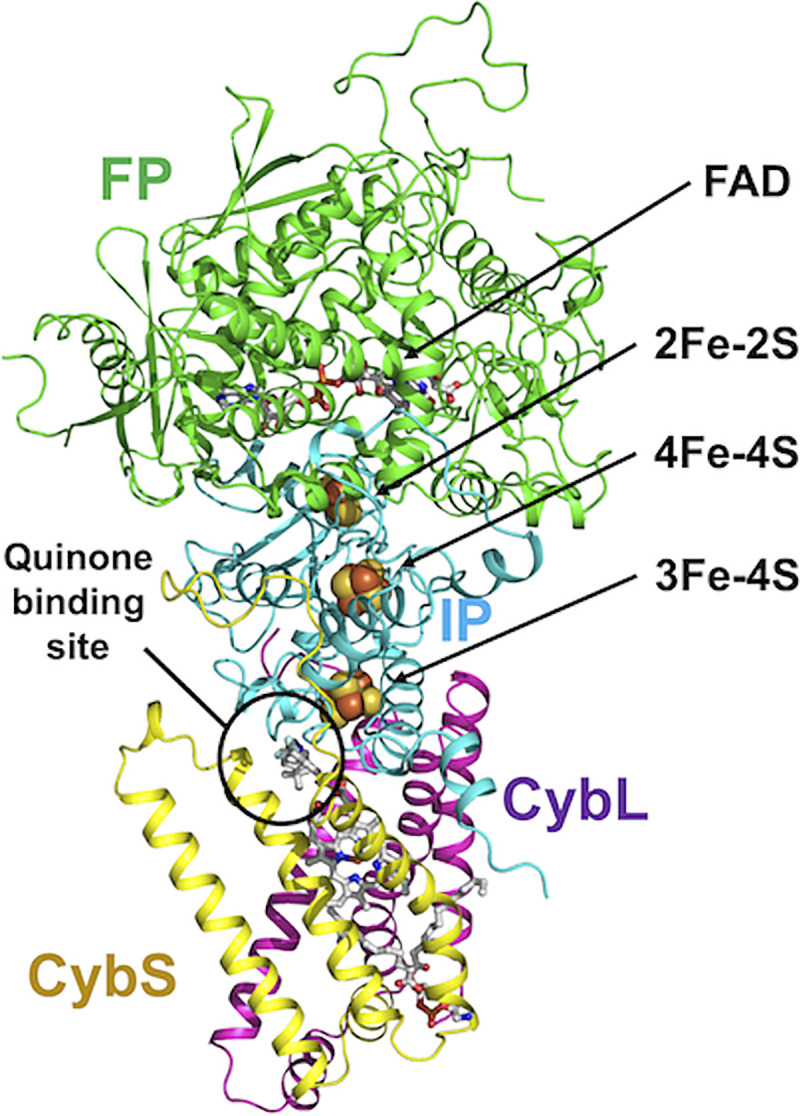
Model structure of E. multilocularis complex II using AlphaFold2. Fp (SDHA), Ip (SDHB), CybL (SDHC), and CybS (SDHD) subunits are colored green, cyan, purple, and yellow, respectively. Four prosthetic groups, FAD, [2Fe–2S], [4Fe–4S], and [3Fe–4S], were also modeled based on the reported crystal structures of *A. suum* and porcine complex II.

**FIG 3:**
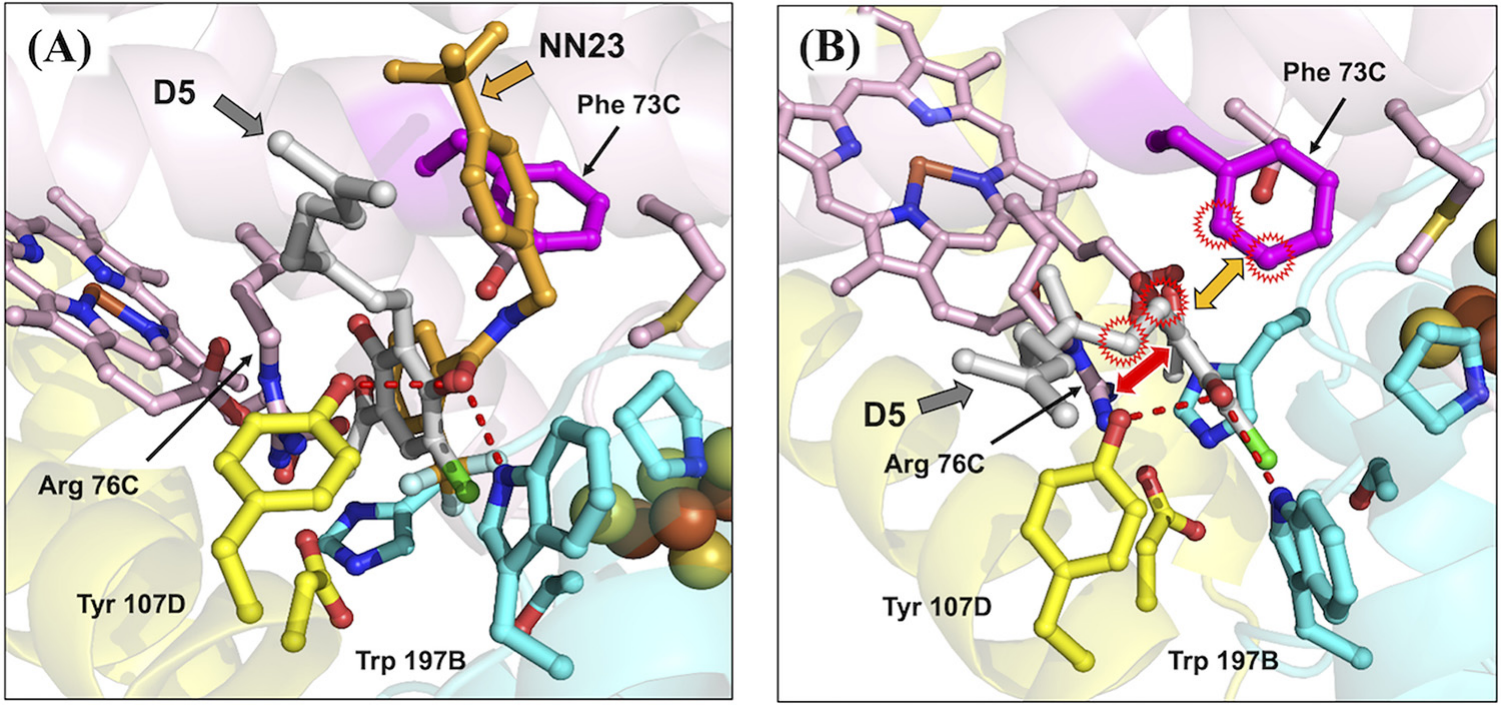
Predicted interaction between AF derivatives and complex II. (A) The position of D5 and a flutolanil derivative (NN23) were merged to predict the interaction between D5 and amino acid residues of E. multilocularis complex II based on the cocrystal structure of *A. suum* complex II with NN23. (B) The 4-OH in D5 may contribute to forming hydrogen bonds with both Tyr107D and Trp197B. Hydrogen bonds are represented as red dotted lines. The red double arrow shows the electrostatic interactions between the guanidino group of Arg76C and the benzene ring of D5. The benzene group of Phe73C, which is not conserved in *A. suum* and porcine complex II, might stabilize the binding of D5 by forming hydrophobic interactions with the linker shown as an orange double arrow and red rings.

A structure prediction of E. multilocularis complex II was performed using AlphaFold2 (Fig. 2). The quaternary structure, including the position of each subunit (Fp, Ip, CybL, and CybS, which correspond to SDHA, SDHB, SDHC, and SDHD subunits, respectively) and prosthetic groups, was based on the crystal structure of *Ascaris suum* complex II that was reported previously by our group (21–23). The predicted structure showed six membrane spanning α-helices (three helices from each of the CybL and CybS subunits) that act as anchors to the mitochondrial inner membrane and amino acid residues surrounding the quinone binding site (Fig. 2). The binding mode of AF derivative D5, the most potent inhibitor of complex II identified in this study, to the quinone binding site formed by Ip, CybL, and CybS subunits was also investigated. The conformation of the bound D5 was predicted based on a previously reported cocrystal structure of *A. suum* complex II with a flutolanil derivative, NN23 (Nihon Nohyaku Co., Ltd., Japan) (Fig. 3A) (22).

**FIG 4:**
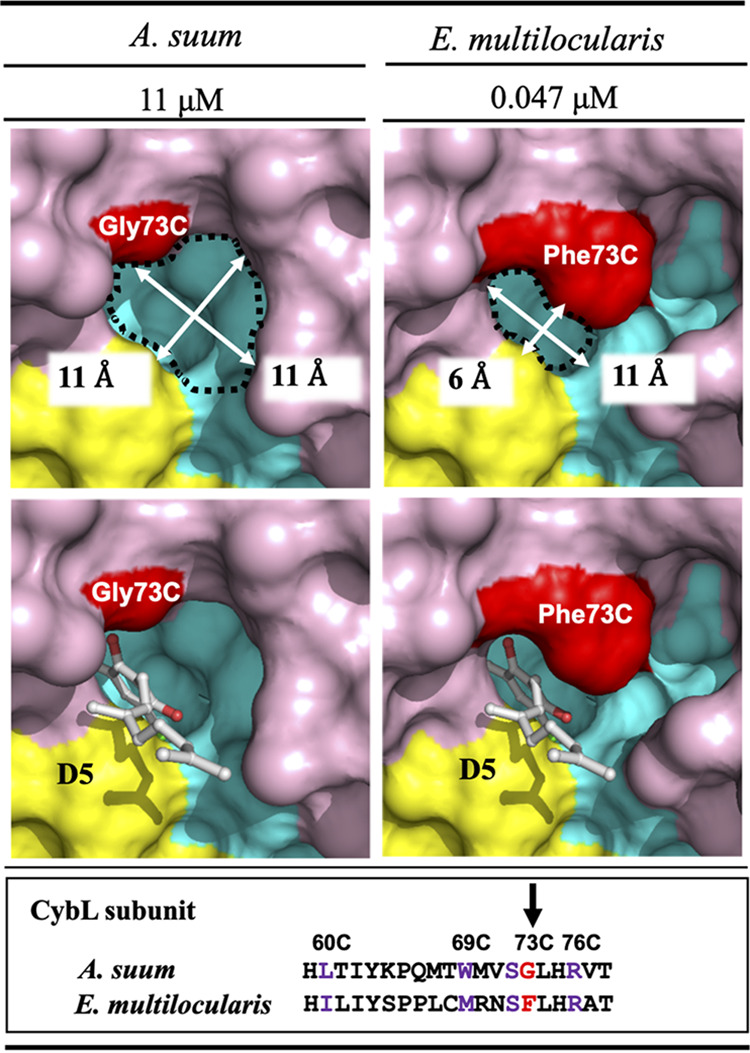
Binding sites of D5 to *A. suum* and E. multilocularis complex II and respective IC_50_ values are denoted. D5 inhibits E. multilocularis complex II (IC_50_ = 0.047 μM) more strongly than that of *A. suum* (IC_50_ = 11 μM). Ip, CybL, and CybS are colored cyan, purple, and yellow, respectively. The Phe73 in CybL and corresponding amino acid residue from *A. suum* are colored red. The dimensions of the entrance to the quinone binding site from *A. suum* and E. multilocularis complex II are 11 × 11 Å (top left panel) and 6 × 11 Å (top right panel), respectively. In the model structure of E. multilocularis complex II, D5 fits neatly into the pocket without steric hindrance with Phe73C (bottom right panel), while a large gap between the benzene group of D5 and pocket inner-surface of *A. suum* complex II can be seen (bottom left panel). The bottom of the figure shows a comparison of the amino acid sequences of CybL from *A. suum* and E. multilocularis. The arrow indicates that Gly73C from *A. suum* is replaced with Phe73C in E. multilocularis.

In E. multilocularis complex II, the entrance of the quinone binding site was narrowed by the presence of Phe73 at the SDHC subunit (Phe73C), which is replaced by glycine (Gly) in the corresponding subunit from *A. suum* complex II. Also, the calculated entrance sizes of the quinone binding sites of *A. suum* and E. multilocularis complex II are 11 × 11 and 6 × 11 Å, respectively. Because of the bulky side chain of the Phe73C residue, our prediction suggests that the quinone binding site of E. multilocularis cannot accommodate NN23 (Fig. S2). These observations are further supported by the lack of inhibition of NN23 against E. multilocularis complex II (NN23 IC_50_>20 μM), although it strongly inhibited the *A. suum* enzyme at an extremely low concentration, NN23 IC_50_ = 0.005 μM (Fig. S1). Conversely, D5 strongly inhibited complex II activity of E. multilocularis with an IC_50_ of 0.047 μM, while it showed 234-fold less potent inhibition (IC_50_ = 11 μM) against *A. suum* complex II activity (Fig. 4). The structure prediction showed that D5 fits tightly into the quinone binding site of E. multilocularis complex II, whereas a large empty space is observed between D5 and the pocket inner surface of *A. suum* complex II (Fig. 4), in accordance with the strong and weak inhibition observed for D5 of the respective enzyme activities. Moreover, D5 may interact tightly with the surrounding amino acid residues. The 4-OH of D5 has the potential to form a hydrogen bond with both Tyr107 from the SDHD subunit (Tyr107D) and Trp197 from the SDHB subunit (Trp197B) (Fig. 3A). In addition, the electron-rich guanidino group plane of Arg76 from SDHC (Arg76C) and the electron-deficient benzene plane ring of D5 are placed in parallel, indicating that they might interact strongly with each other via electrostatic interactions (Fig. 3B) (22). Finally, the isoprene chain from bound D5 may be further stabilized through hydrophobic interactions with the benzene ring of Phe73C, which is not conserved in *A. suum* and mammalian complex II, favoring the binding of D5 to E. multilocularis complex II (Fig. 3B).

### Effects of AF derivatives on the viability of E. multilocularis protoscoleces in culture assay.

**FIG 5:**
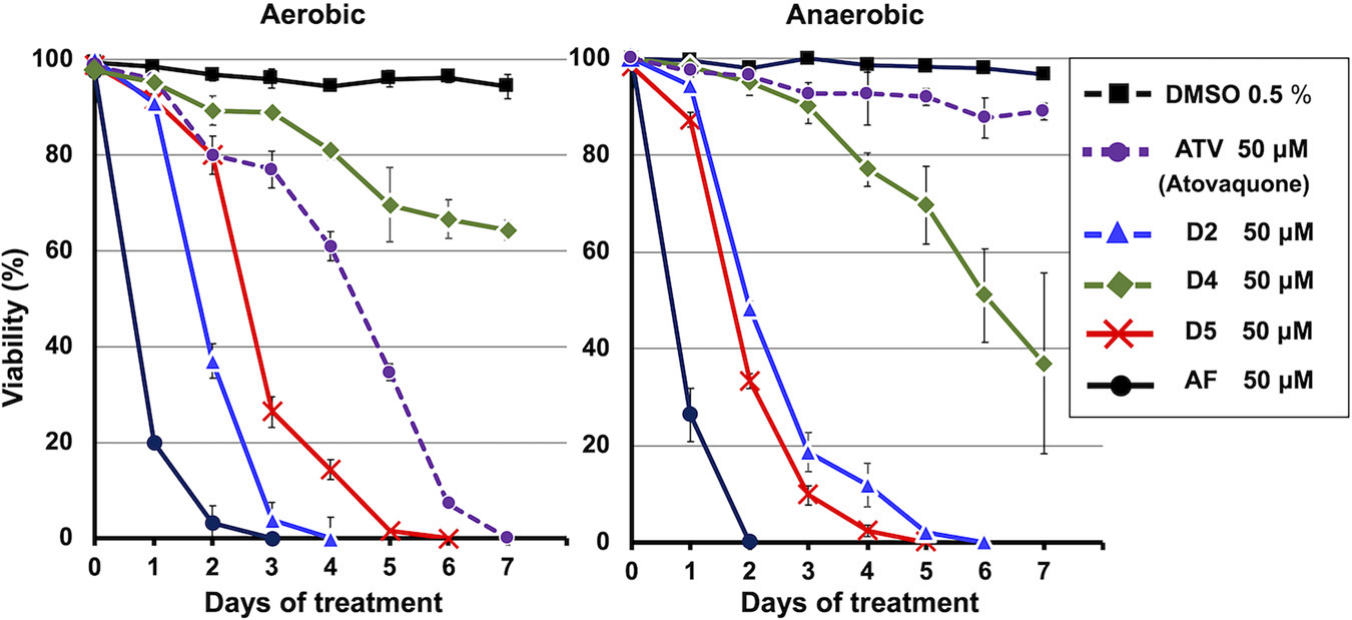
Viability of E. multilocularis protoscoleces in *ex vivo* culture assay. The E. multilocularis protoscoleces were treated with ascofuranone (AF) and its derivatives in culture under aerobic and anaerobic conditions (O_2_ <0.3%). Each compound was added to the culture medium at a final concentration of 50 μM. The control group was supplemented with 0.5% (vol/vol) dimethyl sulfoxide (DMSO). Atovaquone (ATV) was used as positive control and to ensure anaerobic conditions in the culture. The viability of protoscoleces was evaluated by their ability to exclude trypan blue. The data are presented as the mean ± standard deviation (*n* = 3).

A culture assay with protoscoleces was performed under aerobic and anaerobic conditions with AF, D2, D4, and D5. As shown in Fig. 5, the viability of the parasites was progressively reduced during *in vitro* treatment with AF under both aerobic and anaerobic conditions. D2 completely eliminated the parasites by day 4 under aerobic culture conditions, while 6 days were required for complete elimination under anaerobic conditions. D4 showed an antiparasitic effect of only 34% elimination on day 7 under aerobic conditions, whereas 65% of the parasites were eliminated by day 7 under anaerobic culture conditions. D5 eliminated the parasites completely by day 6 and day 5 under aerobic and anaerobic conditions, respectively. Thus, D5 killed the parasites more effectively under hypoxia than normoxia.

## DISCUSSION

In this study, we investigated the effects of AF and its derivatives on the mitochondrial respiratory chain of E. multilocularis. Notably, we demonstrated that AF and its derivatives inhibited both complexes II and III of the mitochondrial electron transport chain at submicromolar to nanomolar levels, showing for the first time that simultaneous inhibition of the fumarate and oxygen respiration with a single compound can be achieved. In addition, several AF derivatives are particularly important because they potently inhibited E. multilocularis complex II, which is directly involved in the anaerobic respiration maintained by parasites inside the host.

The structure-activity relationship of AF derivatives provided important information about the functional groups crucial for inhibition of E. multilocularis complex II, and can be summarized as follows: (i) the 1-acetyl group in the benzene ring improved the inhibition potency compared with a 1-aldehyde group; (ii) the length of the linker required for optimum inhibition was observed to range from C7 to C10, and linkers with two branched methyl groups were preferable; (iii) 5-chlorine is essential for inhibitory activity; and (iv) the 6-methyl group as well as the furanone ring are both dispensable for inhibition; however, depending on the substitution at the terminal group, the inhibitory activity can be increased.

The effect of the AF substituent group structure on enzyme inhibition differs depending on the target. The substitution of the 1-aldehyde group to a 1-acetyl group is deleterious for TAO and HsDHODH inhibitions (15, 24), but seems favorable for inhibition of E. multilocularis complex II. A critical role for the 4-OH group in inhibition seems to be a common trait between TAO, HsDHODH, and E. multilocularis complex II. Because the removal of the 5-chlorine from the benzene group causes the complete loss of inhibition activity, this group might have an essential interactive role through halogen bonding with εN^1^ from Trp197B, similar to what is observed in the cocrystal structures of HsDHODH with AF and its derivatives (15). The 6-methyl group plays an important role in elevating inhibitory activity for the quinone binding site. This group is required for the optimum inhibition of TAO and HsDHODH (15, 24), but it is dispensable for inhibition of E. multilocularis complex II. A plausible explanation is the electron donating nature of the methyl group that induces an electron-rich state of the benzene ring, which reduces the face-to-face parallel interaction with the electron-rich guanidino group of Arg76C, similar to the binding conformation of the trifluoromethylbenzene group of flutolanil derivatives to *A. suum* complex II (22). With respect to the linker group, an unsaturated geranyl linker has been reported to be essential for potent inhibition of TAO (24). However, in E. multilocularis complex II, derivatives with saturated linkers, as in D13, were more efficient than D12. Such kinds of derivatives might bind more flexibly to the quinone binding site than derivatives with a geranyl linker. In addition, the furanone ring has previously been found to be dispensable for the strong inhibition of AF and its derivatives against TAO and HsDHODH, although depending on the terminal group, increased inhibition is observed for HsDHODH (15, 24). Similarly, the inhibitory activity for E. multilocularis complex II was also increased, but with replacement of different terminal groups compared to the derivatives that increased HsDHODH inhibition (15). Derivatives with saturated linkers, and terminal groups such as O-propynyl (D16), might enable the generation of AF derivatives with greater inhibition efficacy against E. multilocularis complex II in the future.

The structure of E. multilocularis complex II, modeled based on the crystal structure of *A. suum* complex II (22, 23), suggested a narrow quinone binding site caused by the bulky side chain of Phe73C. This residue appears to be critical for the target specificity of flutolanil and its derivatives, as it causes steric hindrance with the tert-butylbenzene group of NN23, but not with AF derivatives.

Finally, we assessed the effect of AF and its derivatives on E. multilocularis protoscoleces in a culture assay. We previously hypothesized that a combination of complex II and III inhibitors would inhibit both anaerobic and aerobic respiration, thus killing the parasite (6). Culture assay results for AF, D2, and D5 consistently showed a strong antiparasitic effect on protoscoleces under both anaerobic and aerobic conditions. Although D4 strongly inhibits SQR and succinate-cytochrome *c* reductase, its effect was weak in the culture assay compared to the other derivatives. It is possible that the structure of the compound may affect cell membrane permeability for drug delivery. Comparing the structure of D2 and D5 with D4, the combination of the 1-aldehyde group in the benzene ring and longer side chain may reduce the cell membrane permeability due to the change in the shape of the structure. However, several novel derivatives might be required to more accurately clarify the cause of the difference between D4 and others, which is a limitation of this study.

In the culture assay, the time required to kill protoscoleces in aerobic and anaerobic conditions differed according to the IC_50_ value of each compound. For instance, D4 and D5, which inhibited complex II more potently (IC_50_ = 0.037 and 0.047 μM) than complex III (IC_50_ = 0.109 and 0.221 μM), respectively, showed high killing rates for protoscoleces under anaerobic conditions compared to aerobic conditions. Also, D2 inhibited E. multilocularis complex III with an IC_50_ value of 0.008 μM, whereas the IC_50_ value for complex II was 0.309 μM, which indicates that D2 shows a 38-fold higher selectivity for protoscoleces cultured under aerobic than anaerobic conditions. Interestingly, despite the relatively low inhibitory activity of AF against complex II (IC_50_ = 1.10 μM), it exhibited a strong killing effect under anaerobic conditions. This suggests that the furanone ring as the terminal group of AF might contribute to increased cell membrane permeability; however, further studies are required to confirm this hypothesis.

Our findings demonstrate, for the first time, antiparasitic agents that can potently inhibit both fumarate and oxygen respiration in E. multilocularis. The complete blockage of mitochondrial respiration can lead to a pleiotropic effect in the metabolism of all life cycle stages of E. multilocularis, including the collapse of the mitochondrial electrochemical gradient, ATP synthesis by oxidative phosphorylation (complex V), the NADH oxidation system (complex I), glycerol metabolism (quinone-dependent mitochondrial glycerol-3-phosphate dehydrogenase), and sulfide metabolism (sulfide:quinone oxidoreductase).

Recently, it was reported that simultaneous inhibition of complex III by ELQ-400 (an endochin-like quinolone) and complex I by quinazoline can enhance the effect of ELQ-400 against the metacestodes of E. multilocularis under anaerobic conditions (25). This effect was attributed to the inhibition of aerobic and anaerobic energy metabolism through the inhibition of two respiratory complexes by two different inhibitors. The identification of a single compound simultaneously targeting fumarate and oxygen respiration with potent antiparasitic activity is a unique and groundbreaking feature in the development of novel antiparasitic drugs.

## MATERIALS AND METHODS

### Preparation of the mitochondrial fraction from E. multilocularis protoscoleces.

Cyst tissues containing E. multilocularis (Nemuro strain) protoscoleces isolated from infected hispid cotton rats, Sigmodon hispidus, were used. The tissues were shredded through a metal mesh (0.5 mm diameter). Then, the tissues were repeatedly suspended and washed with physiological saline in a tall beaker to obtain protoscoleces by exploiting the buoyancy difference between protoscoleces and other tissue (26). The obtained protoscoleces were homogenized with a motor-driven homogenizer to prepare the mitochondrial fraction. The homogenate was diluted with mitochondrial preparation buffer (210 mM mannitol, 10 mM sucrose, 1 mM disodium EDTA, and 50 mM Tris-HCl [pH 7.5]) supplemented with 10 mM sodium malonate to 5 times the volume of the original protoscolex homogenate, and then centrifuged at 800 × *g* for 10 min (4°C) to remove cell debris and nuclei. The supernatant was then centrifuged at 8,000 × *g* for 10 min (4°C) to obtain the mitochondrial pellet. Next, the pellet was resuspended in mitochondrial preparation buffer (without malonate) and centrifuged at 8,000 × *g* for 10 min (4°C). The enriched mitochondrial fraction was finally suspended in approximately 0.5 to 1.0 mL of mitochondrial preparation buffer without malonate and frozen at −50°C. The mitochondrial fraction from *A. suum* was prepared essentially as described previously (27, 28).

This study was performed in strict accordance with the National Institutes of Health guide for the care and use of laboratory animals. The ethics committee of the Hokkaido Institute of Public Health approved the protocol for the animal experiments (permit numbers: K26-3, K29-4, and K22-1). All surgeries were performed under anesthesia with sodium pentobarbital and isoflurane, and every effort was made to minimize suffering.

### Enzyme assays and determination of 50% inhibitory concentration (IC_50_).

NADH:quinone reductase activity (complex I), succinate:quinone reductase activity (complex II), and succinate:cytochrome *c* reductase activity (complexes II to III) were measured using a UV-3000 spectrophotometer (Shimadzu, Kyoto, Japan) as described previously (4, 6). Briefly, all enzyme assays using mitochondrial fractions were performed in 0.5- or 1.0-mL reaction mixtures at 25°C. A freeze/thaw process was performed before the assay to ensure that the mitochondrial membrane was permeable to the solutes. The final mitochondrial protein concentration was 50 μg/mL. NADH:quinone reductase activity was measured in 50 mM potassium phosphate buffer (pH 7.4) containing 2 mM potassium cyanide, KCN (Sigma, St. Louis, MO, USA), and 60 μM decylubiquinone (Sigma). The activity assay was started by the addition of 50 μM NADH (Fujifilm-Wako, Tokyo, Japan) and recorded as the rate of NADH consumption as monitored at 340 nm (ε = 6.2 mM^−1 ^cm^−1^). The reagents used in the SQR activity assay were mixed with the reaction buffer containing 50 mM potassium phosphate (pH 7.4) and 0.1% (*wt/vol*) sucrose monolaurate (DOJINDO, Kumamoto, Japan). SQR activity was determined by monitoring the change in absorbance of quinone at 278 nm (ε = 15 mM^−1 ^cm^−1^) in the presence of 60 μM decylubiquinone, 2 mM KCN, and the inhibitor. The reaction was initiated by the addition of 10 mM disodium succinate to the mixture. NADH:cytochrome *c* activity (complex I to III) was measured in the reaction buffer (30 mM potassium phosphate, 1 mM MgCl_2_, pH 7.5) containing 2 mM KCN, 50 μM cytochrome *c* (Nacalai Tesque, Kyoto, Japan), and the inhibitor. The assay was performed after the addition of 100 mM malonate to block complex II activity. The reaction was started by adding 50 μM NADH to the mixture, and the enzyme activity was determined by monitoring the change in absorbance of cytochrome *c* at 550 nm (ε = 19 mM^−1 ^cm^−1^). IC_50_ values of AF and its derivatives against the specific activities of mitochondrial respiratory enzymes in protoscoleces were determined. The IC_50_ of each compound was determined by calculating approximation lines from three or more points on either side of the IC_50_ concentration.

### Computational crystal structure prediction.

We performed protein structure prediction for E. multilocularis mitochondrial complex II using AlphaFold2 (29, 30). The docking of four subunits comprising complex II was based on the crystal structure of *A. suum* and porcine complex II described in previous reports (21–23). The prosthetic group of *A. suum* was included in the final model because AlphaFold2 can only predict polypeptide models. The binding mode of an AF derivative (D5) in E. multilocularis complex II was predicted based on previous reports of the crystal structure of *A. suum* in complex with flutolanil (22, 31, 32). The protein sequences of E. multilocularis complex II subunits were retrieved from the NCBI database using the following accession numbers: Fp (BAX90095.1), Ip (BAX90096.1), CybL (BAX90098.1), and CybS (BAX90099.1).

### Culture assays of live E. multilocularis protoscoleces.

The obtained protoscoleces were cultured in Connaught Medical Research Laboratories 1066 medium (Gibco, Grand Island, NY, USA) containing 0.5% (*wt/vol*) yeast extract (Difco Laboratories, Detroit, MI, USA), 2 mM l-glutamine (Gibco), 23 mM 4-(2-hydroxyethyl)-1-piperazineethanesulfonic acid, 0.5% (*wt/vol*) D (+)-glucose, 0.4 mM sodium taurocholate (Wako Pure Chemical Industries, Osaka, Japan), 57 mM sodium hydrogen carbonate, and 100 U/mL penicillin-streptomycin (Gibco). The protoscoleces were observed daily for seven consecutive days, and half of the medium was replaced on day 3. For anaerobic cultures, six-well plates were sealed in plastic containers with oxygen-detecting agents and oxygen scavengers (Aneromeito, Nissui Pharmaceutical, Tokyo, Japan) to keep the oxygen concentration below 0.3% at 37°C. The protoscoleces were treated with AF and its derivatives at a final concentration of 50 μM in the culture medium, and the duration of parasite elimination was investigated. The control group was supplemented with 0.5% (vol/vol) dimethyl sulfoxide (DMSO), and all conditions were assayed in triplicate. We used ATV in a culture assay as positive control and to ensure anaerobic conditions in the culture (4, 5). The IC_50_ of ATV against SQR and succinate-cytochrome *c* reductase was 0.69 μM and 0.002 μM, respectively. The viability of protoscoleces was determined after microscopic observation of more than 170 protoscoleces per well using the trypan blue exclusion test, as described previously (4, 6, 33).

### Synthesis of AF and its derivatives.

The AF and AF derivatives used in this study were previously designed and synthesized as compounds targeting TAO for anti-trypanosomal drug development (24, 34, 35).

### Statistical analysis.

The results of IC_50_ and culture assay viability determinations are each expressed as the mean ± standard error of the mean (SEM). All statistical analyses were performed using EZR (version R3.6.3; Saitama Medical Center, Jichi Medical University, Saitama, Japan) (36).
