# Supplementary material for: Killing Two Birds with One Stone: Discovery of Dual Inhibitors of Oxygen and Fumarate Respiration in Zoonotic Parasite, Echinococcus multilocularis
Source: Antimicrob Agents Chemother. 2023 Feb 22;67(3):e01428-22. doi: 10.1128/aac.01428-22 (PMC10019194; doi:10.1128/aac.01428-22)
Supplement: Supplemental file 1 — Supplemental material. Download aac.01428-22-s0001.pdf, PDF file, 1.5 MB [file aac.01428-22-s0001.pdf]

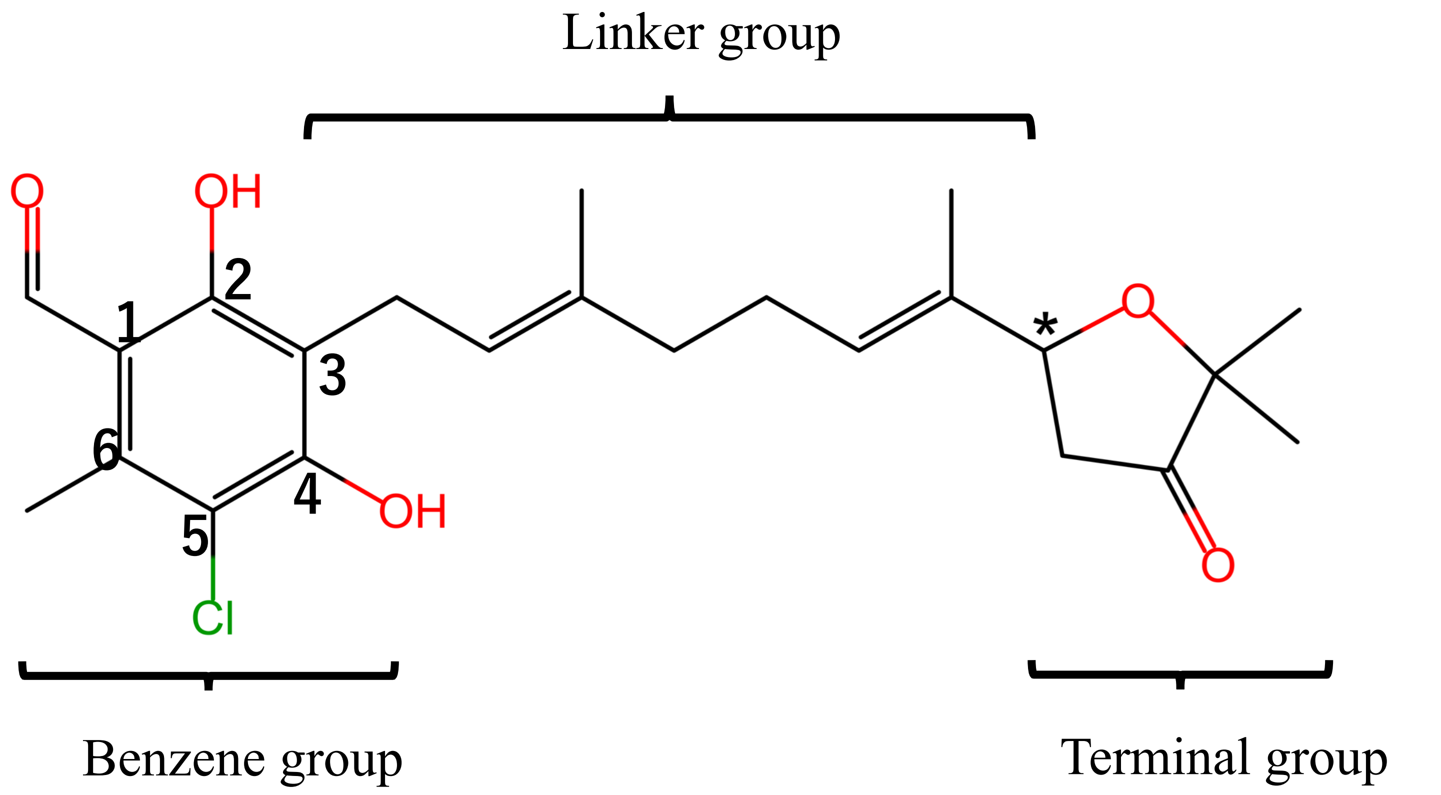

Supplementary Figure 1. Chemical structure of ascofuranone (AF). The benzene, linker, and terminal groups are shown. The asterisk represents the chiral carbon (adapted from Miyazaki *et al.* [1])

NN23 (Flutolanil derivative)

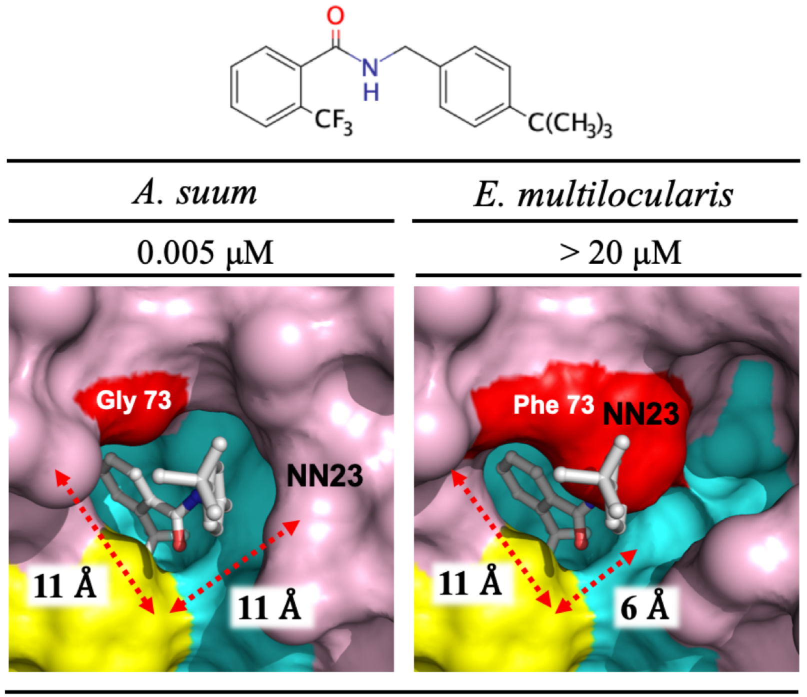

Supplementary Figure 2. Model structure of NN23 bound to the quinone binding site of complex II from *E. multilocularis* based on the reported co-crystal structure (PDB 4YSX) of *A. suum* complex II with NN23. Protein subunits are colored as follows: Ip (cyan), CybL (purple), and CybS (yellow). The dimensions of the entrance to the quinone binding site of *A. suum* and *E. multilocularis* complex II are shown as in Figure 4. As rendered in ball-and-stick, NN23 binds tightly to *A. suum* complex II (IC_{50} = 0.005 μM), but it did not inhibit *E. multilocularis* complex II (IC_{50} > 20 μM) due to the steric hindrance between Phe73C and the tert-butylbenzene group of NN23.

References

[1] Miyazaki Y, Inaoka DK, Shiba T, Saimoto H, Sakura T, Amalia E, Kido Y, Sakai C, Nakamura M, Anthony L Moore, Harada S, Kita K. 2018. Selective Cytotoxicity of Dihydroorotate Dehydrogenase Inhibitors to Human Cancer Cells Under Hypoxia and Nutrient-Deprived Conditions. Front Pharmacol 9:997.
